# Evaluation and Implementation of a Proactive Telephone Smoking Cessation Counseling for Parents: A Study Protocol of an Effectiveness Implementation Hybrid Design

**DOI:** 10.3390/ijerph15010097

**Published:** 2018-01-09

**Authors:** Tessa Scheffers-van Schayck, Roy Otten, Rutger Engels, Marloes Kleinjan

**Affiliations:** 1 Trimbos Institute, Netherlands Institute of Mental Health and Addiction, P.O. Box 725, 3500 AS Utrecht, The Netherlands; rengels@trimbos.nl; 2 Department of Psychology, Utrecht University, P.O. Box 80140, 3508 TC Utrecht, The Netherlands; 3 Department of Research and Development, Pluryn, P.O. Box 53, 6500 AB Nijmegen, The Netherlands; royotten@pluryn.nl; 4 ASU REACH Institute, Department of Psychology, Arizona State University, P.O. Box 876005, Tempe, AZ 85287-6005, USA; 5 Department of Cultural Diversity & Youth, Utrecht University, P.O. Box 80140, 3508 TC Utrecht, The Netherlands

**Keywords:** smoking cessation, parents, children, telephone counseling, proactive recruitment, implementation channels, effectiveness-implementation hybrid design

## Abstract

Detrimental health consequences of smoking for both parents and children stress the importance for parents to quit. A Dutch efficacy trial supported the efficacy of proactive telephone counseling on parents. Still, how this program would function in “real world” conditions and how parents could be optimally reached is unclear. Therefore, this study will use an innovative method to examine the recruitment success of two implementation approaches (i.e., via a healthcare approach and a mass media approach) to test the (cost)effectiveness of the program. A two-arm randomized controlled trial and an implementation study (i.e., process evaluation) are conducted. Parents (*N* = 158) will be randomly assigned to the intervention (i.e., telephone counseling) or control conditions (i.e., self-help brochure). Primary outcome measure is 7-day point prevalence abstinence at three months post-intervention. Qualitative and quantitative research methods are used for the process evaluation. We expect that parents in the intervention condition have higher cessation rates than parents in the control condition. We also expect that the recruitment of parents via (youth) health care services is a more promising implementation approach compared to mass media. Results will have implications for the effectiveness of a proactive telephone counseling and provide directions for its successful implementation.

## 1. Introduction

Cigarette smoking continues to be the most preventable cause of mortality and morbidity [1]. In the Dutch population of 17 million people, close to 19,200 people died from smoking-related diseases (e.g., lung cancer and Chronic Obstructive Pulmonary Disease (COPD)) in 2014 [2]. In addition, in 19% of Dutch families with children younger than 18 years, children are exposed to indoor smoking [3]. Exposure to Environmental Tobacco Smoke (ETS) has detrimental effects on children’s physical health, including increased incidences of middle ear disease, reduced lung function, and increased frequencies of childhood asthma and bronchitis [4,5]. It is important that parents quit smoking to eliminate the majority of the children’s ETS [6] and diminish the risk for children to start smoking [7,8]. Moreover, it reduces the odds of morbidity and mortality for smoking parents themselves [9].

The existing research suggests that most parents want to quit smoking and even tried to quit smoking [10]. In addition, most parents would accept smoking cessation support, such as telephone counseling [11]. A review consisting of 77 (quasi) randomized controlled trials (RCT) on telephone counseling confirmed that telephone counseling is effective in increasing smoking cessation rates [12]. Moreover, Hollis et al., 2007 [13] found that telephone counseling is cost-effective (i.e., the degree to which something is effective in relation to its costs). Recently, a tailored telephone counseling program for smoking parents was tested in The Netherlands [14]. Parents who smoke (*N* = 512) were recruited in an RCT and assigned to the intervention condition (i.e., tailored telephone counseling and supplementary materials) or the control condition (i.e., a standard self-help brochure). Parents who received telephone counseling were more likely to report 7-day point prevalence abstinence at three months post-intervention (44.5% versus 12.1%; OR = 6.89, 95% CI = 4.18–11.36) and to use nicotine replacement therapy (48.4% versus 20.9%) compared to parents who received a standard self-help brochure. Among parents who did not quit smoking, those who received telephone counseling were more likely to make a quit attempt (85.2% versus 68.1%) and to implement a complete home smoking ban (39.5% versus 26.1%) compared to parents who received the self-help brochure. Altogether, this study provided evidence that telephone counseling tailored to smoking parents is effective in helping parents quit smoking and developing parenting practices that protect their offspring from ETS exposure [14].

Although Schuck et al., 2014 [14] found that the telephone counseling was effective, they tested it in an efficacy trial. This means that the telephone counseling was evaluated under optimum conditions [15]; the counseling was offered for free, and participating parents were rewarded with 100 euros after completing the study. Therefore, how the telephone counseling would function when examined in “real world” conditions (i.e., in an effectiveness trial) is unclear. Moreover, despite its effectiveness, intervention success also depends on the extent to which people have access to the program and the acceptance and utilization of these programs [16]. An important predictor of the reachability of smoking cessation programs concerns their recruitment strategy [17]. Recruitment strategies can be classified into proactive recruitment strategies (i.e., people are contacted directly, and services are offered to them) and reactive recruitment strategies (i.e., people are informed about the availability of an intervention and those who are interested have to volunteer to participate) [18]. Many programs use a reactive recruitment strategy [19], which could explain why only a minority of smokers use smoking cessation programs [20]. In contrast, proactive recruitment strategies have proven to be effective in improving the reachability of smoking cessation programs [16,21,22] and in promoting smoking cessation among smokers [23,24,25].

In addition to recruitment strategies, recruitment venues are also important aspects of the reachability of smoking cessation programs. In the Dutch efficacy study, primary schools were used to recruit smoking parents for a telephone counseling [22]. According to the authors, primary schools can reach the majority of smoking parents and function as a ‘teachable setting’ for smoking parents [22]. The results of this study have shown that the primary schools are willing to promote telephone smoking cessation counseling to parents. In addition, this recruitment channel resulted in relatively low recruitment costs (21.74 euros per parent) and a high reach (approximately 10,000 households). However, despite the low costs, the response rate was low (5%), even though participating parents were rewarded with 100 euros after completing the study. The effect of the financial reward of 100 euros for the participants’ willingness to participate on the response rate of smoking parents of children in primary schools is unclear. Further research is needed to gain insight into whether primary schools are a good implementation channel to connect smoking parents with a proactive telephone smoking cessation counseling.

Online mass media (e.g., smoking cessation websites and social media) is another promising channel that could connect parents who smoke with a proactive telephone counseling. In The Netherlands, smokers who are interested in smoking cessation can visit various smoking cessation websites, including the national online smoking cessation website (i.e., www.ikstopnu.nl). This website, funded by the Dutch government, had 89,116 unique visitors in 2016 [26]. Some studies have provided evidence that online mass media can be successful and cost-effective in recruiting smokers for cessation support [27,28,29]. However, to our knowledge, little is known about whether online mass media is successful in connecting parents who smoke to a proactive telephone counseling. Hence, further research is needed to assess the implementation of this program.

In addition to a mass media approach, the health care setting can also be suitable for addressing parental smoking [30,31,32]. Winickoff and colleagues (2001) [33] found that 74% of smoking parents whose children are hospitalized would enroll in a free telephone counseling cessation program. Moreover, these parents are also more motivated to quit smoking, since they worry more about the consequences of their smoking for their children’s respiratory diseases [34]. In The Netherlands, youth health care could be an ideal setting to address parental smoking cessation, as the Dutch youth health care provides preventive health care to all children (aged 0 to 17 years old) [35]. In The Netherlands, all parents must visit youth health care centers 15 times in the first four years of their child’s life [35]. In addition, youth health care professionals are expected to address parental smoking (cessation) at least four times during that time, if applicable [36]. Research shows that it is considered to be the (youth) health care professional’s role to address parental smoking [37,38,39]. However, (youth) health care professionals experience several barriers in doing so (e.g., a lack of time, the possibility of damaging the doctor-patient relationship, and lack of confidence in own smoking cessation counseling skills) [38,39,40]. Therefore, it is essential to provide a convenient, user-friendly, and time-saving intervention to (youth) health care professionals that does not jeopardize the doctor-patient relationship. In the present study, a time-saving and convenient intervention will be provided that allows (youth) health care professionals to easily refer smoking parents to a telephone counseling (see the methods section for an elaborate description of our intervention).

In sum, this effectiveness study takes a novel method by examining the recruitment success of two implementation approaches (i.e., a mass media approach and a health care approach) to test the (cost)effectiveness of a telephone smoking cessation counseling and whether the effectiveness depends on the implementation approach. The mass media approach includes two implementation channels: mass mailings through primary schools and online mass media. The health care approach also consists of two implementation channels: general health care and youth health care. A 2-arm RCT will be carried out to test the (cost)effectiveness of the telephone counseling. Based on earlier results of Schuck et al., 2014 [14], it is expected that parents who receive telephone counseling show higher smoking cessation rates at 3-month post-intervention compared to parents who receive the control condition. Concerning the implementation of the telephone counseling, a process evaluation of the recruitment success will be conducted for two implementation approaches. It is expected that (youth) health care will be a more promising implementation approach for connecting smoking parents with the telephone counseling compared to mass media channels (online approach and mass mailings trough primary schools), as most of these parents are motivated to quit and find it acceptable to be directed to telephone counseling in the context of their child’s health [11,34].

## 2. Materials and Methods

### 2.1. Study Design

**Figure 1:**
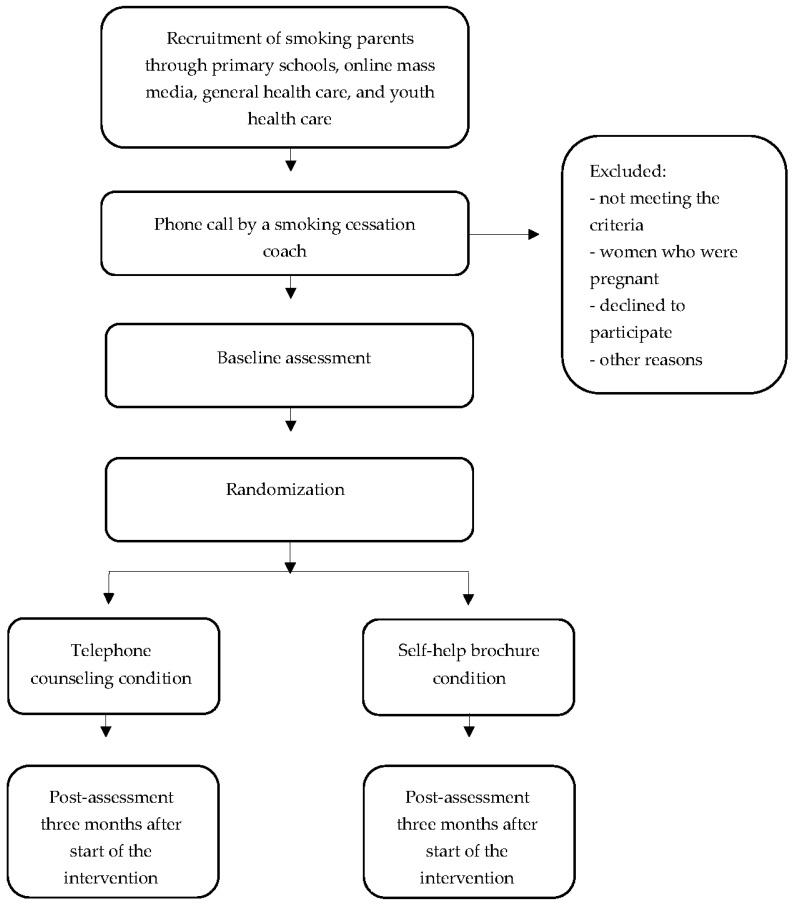
Study design.

Because both the (cost)effectiveness and the implementation strategy of the telephone counseling will be examined, an effectiveness-implementation hybrid design will be adopted. This design has multiple advantages, including improving the capability to identify important implementation strategies and interactions, increasing the speed of implementing the interventions once deemed effective, and providing valuable information for decision makers [41,42]. With respect to the RCT, parents who smoke will receive two online questionnaires during a period of three months. In total, 158 smoking parents will be randomly assigned to the intervention (i.e., telephone counseling) or control (i.e., self-help brochure) condition. The study design is depicted in Figure 1.

### 2.2. Participants and Recruitment

In total, 158 smoking parents will be recruited through four different implementation channels (i.e., primary schools, online mass media, health care, and youth health care), which will be distinguished as two different implementation approaches (mass media approach and health care approach).

#### 2.2.1. Mass Media Approach

##### Primary Schools

A random sampling strategy will be used to recruit smoking parents through their children’s primary schools across all 12 provinces of The Netherlands. The randomly selected primary school boards will be asked to (1) distribute invitation letters to all children (aged 4–12 years) and ask children to hand these letters to their parents or (2) send the letters directly to the parents via e-mail. The primary schools decide which option they prefer. The invitation letters, which are targeted on parents in general and not personalized, will include a description of the study’s purpose, frequency of the assessments, and inclusion criteria. Smoking parents recruited via primary schools will be able to register for the study by completing the registration form (active informed consent) on the study website or by returning the form via mail. After the completion of the registration form, the parents will be called by professional counselors of SineFuma (one of the Dutch certified quit lines). During this phone call conversation, parents will receive additional information about the study (e.g., parents who will be randomized to the telephone counseling might have to pay for the telephone counseling (based on the parents’ health insurance), whereas parents who are randomized to the control condition do not have to pay for their intervention) based on which they will either confirm or withdraw their registration.

##### Online Mass Media

Parents will be recruited through social media and two smoking cessation websites, including the Dutch national smoking cessation website. With respect to social media, multiple paid Facebook advertisements will be created and used for several months. The advertisements will target on parents who are interested in smoking (cessation). Both format and message of the advertisements can differ, depending on what message and/or format will be more successful in recruiting parents who smoke. Whether advertisements are successful or not will be based on the click through ratio (i.e., the ratio of users who click on the Facebook ad of the total users who view the ad) and the costs per click on the ads. Parents who will be recruited through online mass media have to complete a short registration form (i.e., name, telephone number, and e-mail address) on the website of SineFuma so that they can be called by the coaches within one week. Parents who will fulfill the inclusion criteria will be given a description of the study. Parents who will not fulfill the inclusion criteria will not be included in the study, but they will be offered telephone counseling. Parents who will be willing to participate in this study will receive a registration form and will be asked to return the completed form (active informed consent) by mail or online on the study website.

#### 2.2.2. Health Care Approach

General health care and youth health care professionals (henceforth referred to as health care professionals) will be recruited using multiple methods, e.g., word of mouth among health care professionals and social media (i.e., Facebook and LinkedIn). Participating health care professionals will receive materials to be used for support. When smoking parents come to see a health care professional, they will be asked whether they want to receive effective smoking cessation support and whether they want a smoking cessation coach to call them. When parents give their permission, health care professionals will be able to register them online by telephone or by fax. Subsequently, professional counselors of SineFuma will contact these parents within one week. The registration process for the study will be identical to the registration process through online mass media.

### 2.3. Inclusion Criteria

To participate in the RCT, parents will have to: (1) be a parent/caretaker of a child between 0 and 18 years old; (2) be at least a weekly smoker; (3) have the intention to quit smoking currently or in the future; and (4) give informed consent to participate. Pregnant women will be excluded, and telephone counseling will be offered. The ethics committee of the Trimbos Institute has approved this study’s protocol (201607_52-1606).

### 2.4. Study Conditions

After giving informed consent and completing the baseline assessment, parents will be randomly assigned to one of the two trial conditions, telephone counseling condition or control condition.

#### 2.4.1. Telephone Counseling Condition

In the telephone counseling condition, parents will receive up to six proactive counseling phone calls (20 min) over a period of 10 weeks. The telephone counseling is based on Motivational Interviewing (MI), which is “a directive, client-centered counseling style for eliciting behavior change by helping clients to explore and resolve ambivalence” [43]. It aims to motivate patients to change, to focus on the changes that are most important to them, and to focus on their strengths instead of weaknesses and problems [44]. MI has been widely used for smoking cessation programs, and it has proven effective for smoking cessation support [45,46].

The telephone counseling will be performed by professional smoking cessation counselors of SineFuma who are thoroughly trained, experienced, and certified in delivering telephone counseling to support smoking cessation. During the six calls, multiple topics will be discussed, including information about nicotine replacement therapy, personal motivation and smoking history, preparation for the quit date, withdrawal symptoms, craving, relapse prevention, weight gain, and having a smoke-free future. Since the smoking cessation coaches will book individual appointment with parents at times that suit them, uptake will be maximized and drop-outs will be minimized. The results of Schuck et al., 2014 [14] showed that in the telephone counseling condition 224 parents (87.5%) indicated receiving at least one counseling call and 212 (82.8%) indicated receiving at least three calls. Of parents who received calls, the mean number of calls received was 5.5 (SD = 1.8). In addition, all parents will receive a supplementary brochure on smoking cessation, which was designed for this study as supplementary material for parents. The brochure (Rookvrije Ouders (Smoke-free Parents)) consists of 22 pages (color-printed, size: 30 × 21 centimeters) and provides didactic information about smoking that is relevant to parents (e.g., information about ETS) as well as exercises, motivational messages, and tips that are relevant to parents who want to quit smoking. To guarantee quality and comprehensibility of the brochure, professional counselors of SineFuma and communication experts were involved in the development of the brochure. Parents will receive this brochure by mail immediately after the start of the telephone counseling.

#### 2.4.2. Control Condition

Parents in the control condition will receive a self-help brochure on smoking cessation within one week after completing the baseline assessment. The brochure (‘Wat je zou moeten weten over stoppen met roken’ (‘What you should know about smoking cessation’)) is a 16-page, color-printed booklet (size: 30 × 21 centimeters) developed by the Trimbos Institute. The brochure includes elements that have shown to be effective, such as using positive formulation and focusing on the benefits of smoking cessation. The brochure is divided into nine parts: information about smoking and smoking addiction, deciding to quit smoking, consequences of smoking, advantages of smoking cessation, tips and exercises, available smoking cessation methods, pharmacotherapy, maintenance of smoking cessation, and physical symptoms associated with quitting smoking. Parents in the control condition will be able to receive the telephone counseling at the end of the study (i.e., when parents have completed the second assessment).

### 2.5. Data Collection

The timing of the data collection will be different for each implementation channel. Parents will be recruited gradually from September 2016 to September 2018. This means that the data of the two assessments will be obtained at different moments during the data collection (i.e., at the start of the study and at 3 months after the start of the intervention). All parents will receive a personal invitation by e-mail to complete two questionnaires online at a secured website. The web survey software application Jambo (January 2017) will be used to collect parents’ answers. At the end of the study, multiple family excursions will be raffled among parents (e.g., free entry permits for an amusement park). The parents will be told that the raffles will not be part of the parents’ incentives to enroll in the study, but to complete the two online questionnaires.

### 2.6. Randomization

An independent member of the research group will conduct randomization using a computer-generated allocation sequence. Parents will be stratified by educational level (i.e., low: no high school diploma/no vocational training; medium: vocational training or high school diploma; high: college degree), implementation channel (i.e., primary schools, health care, youth health care, and online mass media), and number of cigarettes smoked per day (i.e., less than 10; 10–20; 21 or more). When both parents live in the same household and participate in this study, randomization will be carried out at household level to avoid contamination between the two conditions.

### 2.7. Sample Size

Based on the results of Schuck and colleagues (2014) [14], our power calculation (Stata 12.1, StataCorp., College Station, TX, USA) indicated that 72 parents per implementation approach (36 intervention and 36 control) is sufficient to detect 7-day prevalence abstinence at three months post-intervention with a large effect (*p*_1_ = 0.445, *p*_2_ = 0.121, Power = 0.80, α = 0.05). Because we want to examine differences in smoking cessation rates between the two implementation approaches, we have to double from 72 to 144 parents. Based on the drop-out rates at 3-month follow-up of Schuck et al., 2014 [14] (i.e., 10% in the control group and 3.9% in the intervention group), we will anticipate 10% drop-out. This means that we will aim to recruit a minimum of 158 participating parents in total.

### 2.8. Outcomes

The primary outcome measure will be 7-day point prevalence abstinence at three months post-intervention. In addition, secondary outcome measures include: (1) occurrence of at least 24 h of abstinence at three months post-intervention; (2) increase in motivation to quit; (3) number and duration of quit attempts; (4) use of and adherence to nicotine replacement therapy; (5) implementation of smoking restrictions at home; and (6) change in smoking-related cognitions (e.g., social norms, attitudes towards smoking, and self-efficacy). Moreover, to compare the success rates in this study with other studies, one outcome of the Russell Standard (Clinical) version 2 [47] will also be included as a secondary outcome (i.e., 14-day point prevalence abstinence at 4 weeks after the designed quit date).

### 2.9. Economic Evaluation

The present study examines the cost-effectiveness of the telephone counseling in accordance with the CHEERS checklist [48]. Four types of costs will be considered: (1) the costs of offering the interventions; (2) recruitment costs for each implementation approach and channel; (3) costs stemming from health care uptake; and (4) costs stemming from losses in productivity, in both paid work and volunteer jobs. Costs of the interventions and implementation channels and approaches (e.g., sending materials to health care professionals and advertisements on Facebook) will be monitored during the study. Other costs will be calculated by using standard cost prices (for reference year 2014), which are published in the Dutch guideline for costing [49]. The data on costs stemming from health care uptake and losses in productivity will be collected using the Trimbos/iMTA Questionnaire on Costs associated with Psychiatric illness (TiC-P) [50]. The TiC-P is the most widely used patient-reported measure of health service utilization in The Netherlands [51]. The cost-effectiveness analyses (CEA) will be conducted along the RCT, with 7-day point prevalence abstinence at three months post-intervention as the primary outcome (i.e., the incremental effect). The incremental costs will be calculated as the average cost difference between the conditions. Both the incremental effect and incremental costs will be combined to compute the incremental cost-effectiveness ratio (ICER; incremental costs/incremental effects). To manage stochastic uncertainty, 2500 non-parametric bootstraps will be used and the bootstrapped ICERs will be plotted on the ICER plane. Based on the four quadrants of the ICER plane, four scenarios are possible regarding the telephone counseling, which can: (1) costs more and be more effective; (2) costs more and be less effective; (3) costs less and be less effective; and (4) costs less and be more effective. As seen from a health-economics perspective, the second and fourth scenarios are clear cut cases for decision-makers, with the second scenario being the least attractive (and the intervention is dominated by its alternative (the self-help brochure)) and the fourth scenario being the most attractive, and decision-makers will opt for the intervention. On the other hand, the first scenario is more difficult for decision-makers. In this scenario, one needs to decide how much one is willing to pay for an additional unit of effect (one extra parent who was successful in refraining to smoke) [52]. To facilitate this decision, an ICER acceptability curve will be plotted with the probability that one deems the intervention to be cost-effective (on the *y*-axis) against various willingness-to-pay ceilings (on the *x*-axis). Finally, the CEA will be completed by performing one-way sensitivity analyses directed at uncertainties in major cost-drivers. This is done to ascertain the robustness of the CEA findings.

### 2.10. Statistical Analyses

Statistical analyses will be carried out to assess whether the randomization results in an equal baseline distribution of relevant participant characteristics across conditions. In case of group differences at baseline, confounding variables will be included in subsequent analyses. All analyses will be conducted in accordance with the intention-to-treat principle (i.e., all parents randomized to one of the two conditions will be included in the analyses examining the study hypotheses) as per the Consolidated Standards of Reporting Trials [53]. Moreover, a completers-only analyses will be conducted, i.e., only parents with outcome data on all assessments will be included in the analyses. To compare cessation rates across different groups, logistic regression analyses will be conducted. In addition, Mplus will be used to test for moderation and subsequently, multi-group testing with bootstrapping will be conducted to assess differences between recruitment approaches. Effect sizes and 95% confidence intervals (CI) will be reported to determine the magnitude and effect of the intervention.

### 2.11. Process Evaluation

Our process evaluation will examine: (1) the recruitment success (i.e., the number of parents who signed up to be called by a smoking cessation coach divided by the number of parents who started the intervention) of the four implementation channels (i.e., primary schools, online mass media, general health care, and youth health care) as well as the overarching approaches (mass media and healthcare); (2) the number of the counseling phone calls parents received and whether this differs between the four implementation channels and overarching approaches; (3) the facilitators of and barriers to participation of parents and professionals in the implementation channels (e.g., whether the costs of the telephone counseling keep parents from receiving the counseling); and (4) parents’ experiences, opinions, and perceptions with regard to the telephone counseling (e.g., whether parents have suggestions for improvement of the counseling and whether there were any topics that were missing and should have been discussed during the sessions). In accordance with Linnan and Steckler (2002) [54] multiple important process evaluation components will be included, such as fidelity (e.g., whether health care professionals follow the recruitment protocol), reach (e.g., the extent to which parents were reached by the different implementation channels), and context (e.g., factors that affect the recruitment or telephone counseling). Questionnaires, semi-structured interviews, and focus groups will be used to collect the data at a process level.

## 3. Discussion

This study protocol describes the hybrid design of an effectiveness-implementation study that takes an innovative method by examining the recruitment success of two implementation approaches (i.e., a mass media approach and a health care approach) to test the (cost)effectiveness of a proactive telephone counseling program for smoking parents of children aged 0–18 years. By evaluating the different implementation approaches and their effect on the (cost)effectiveness, this study aims to provide more insight into how this smoking cessation program can be efficiently and sustainably implemented in The Netherlands. Based on earlier results of Schuck et al., 2014 [14], it is hypothesized that the cessation rates of parents who receive telephone counseling will be higher at three months post-intervention compared to the cessation rates of parents who receive a self-help brochure. In addition, it is expected that (youth) health care professionals will be a more promising implementation approach for connecting smoking parents with the telephone counseling compared to (online) mass media because most parents are motivated to quit and find it acceptable to be directed to telephone counseling when their child’s health is concerned [11,34].

### Strengths and Limitations

A strength of this study is that the Russell Standard (Clinical) version 2 [47] will be used to assess smoking cessation. It provides standard criteria of successful smoking cessation rates to enable meaningful comparisons between different studies [47]. Another strength is that as an effectiveness and an implementation study, it allows for the identification of important implementation strategies and interactions, offering valuable information to decision-makers [41,42]. In addition, a mixed-method design (i.e., quantitative and qualitative research) yields stronger evidence because of convergence and confirmation of the results [55]. In accordance with Schuck et al., 2014 [14] some secondary outcomes will be included. However, our power calculation was only based on the primary outcome. Another limitation is that we will not be able to assess smoking cessation rates with a long term assessment (e.g., one year follow up). However, in accordance with Schuck et al., 2014 [14] we have a follow-up assessment at three months. In addition, we will use the Russell Standard (Clinical) version 2 [47] to assess smoking cessation rates. Therefore, we are able to compare our smoking cessation rates with that of other studies. Finally, smoking cessation will be measured using self-reports, which may lead to an under-reporting due to the social desirability bias [56]. To verify this potential bias, we will inform parents in the introduction of the second questionnaire that a random subsample of parents (30%) will be approached for biochemical validation (NicAlert dipstick (Nyomax, Hasbrouck Heights, NJ, USA).

## 4. Conclusions

The results of this study will reveal whether the telephone counseling is effective in “real life” conditions. If the counseling will not be as strong as we expect we will search for reasons and try to clarify why our results do not correspond to the results of Schuck et al., 2014 [14]. Consequently, we will think about how the telephone counseling can be improved. The results of Hayes et al., 2017 [57] could be important and relevant suggestions for the improvement of the telephone counseling. This study will also provide information on how and where to implement the telephone counseling for smoking parents in the most optimal manner in The Netherlands. If all four implementation channels will turn out successful in recruiting smoking parents (i.e., high recruitment rates and similar costs/participant), we will be able to conclude that a combination of all four implementation channels should be used to implement the telephone counseling. In contrast, if only one implementation channel turns out to be unsuccessful (i.e., extremely low recruitment rates and/or low quit rates), this particular channel should not be used to implement this program.
